# The Magnetic and Crystal Structure of Mn_x_Ga (1.15 ≤ x ≤ 1.8) Alloys

**DOI:** 10.1038/s41598-017-00579-w

**Published:** 2017-04-05

**Authors:** D. H. Ryan, Ming Yue, C. B. Boyer, X. B. Liu, Qingmei Lu, Hongguo Zhang, Chenhui Li, Manli Wang, Z. Altounian

**Affiliations:** 1 Physics Department and Centre for the Physics of Materials, McGill University, 3600 University Street, Montreal, Quebec H3A 2T8 Canada; 2 College of Materials Science and Engineering, Beijing University of Technology, Beijing, 100122 China; 3 Canadian Neutron Beam Centre, Chalk River Laboratories, Ontario, K0J 1J0 Canada

## Abstract

Neutron powder diffraction patterns measured above T_*C*_ have been used to determine the location of the excess Mn in Mn_x_Ga (1.15 ≤ x ≤ 1.8). This information has then been used to constrain the fits to neutron powder diffraction patterns measured at ambient temperature and so determine unambiguously the Mn moments in this system. We find that Mn randomly occupies the two Ga sites (2*a* and 2*b*) in the I4/*mmm* structure and propose that it is more appropriate to use a simpler structure based on the P4/*mmm* space group with a reduced unit cell. In this structure the two Ga sites are formally equivalent (they occupy the 1*a* site while Mn occupies the 1*d* site). Our experimental observations are supported by DFT calculations. Below T_*C*_ we find that the Mn(1*d*) moment is constant at 2.45(3) *μ*_*B*_, while Mn on the 1*a* site carries a slightly larger moment (~3 *μ*_*B*_) that is coupled antiparallel to the Mn(1*d*) moments, leading to the observed drop in magnetisation with increasing Mn content in Mn_x_Ga.

## Introduction

Manganese-based magnetic compounds are of interest for two main reasons: (1) they avoid the use of rare earths, (2) they offer modest-cost/modest performance alternatives to the two dominant hard magnet technologies: Nd_2_Fe_14_B (515 kJm^−3^) and Ba(Sr)Fe_12_O_19_ (45 kJm^−3^)^1^. Mn frequently carries a large moment, has reasonable chemical stability, and comes at a moderate cost. One possible system is Mn-Ga. The predicted saturation magnetisation of stoichiometric MnGa is 116 J/T/kg^2^ and if a reasonably square loop could be achieved, this would yield an energy product in excess of 200 kJm^−3^. Unfortunately, the Mn-Ga binary phase diagram is extremely complex, lacking a single congruently-melting compound. As a result, sample preparation is complex and quality is often poor. The problem has been compounded by focusing on producing technically interesting materials before the intrinsic properties have been understood.

In a previous paper we described the excellent intrinsic magnetic properties of the Mn_x_Ga system^3^. Here we concentrate on establishing the basic crystal and magnetic structures; determining the location and ordering behaviour of the excess manganese.

**Figure 1:**
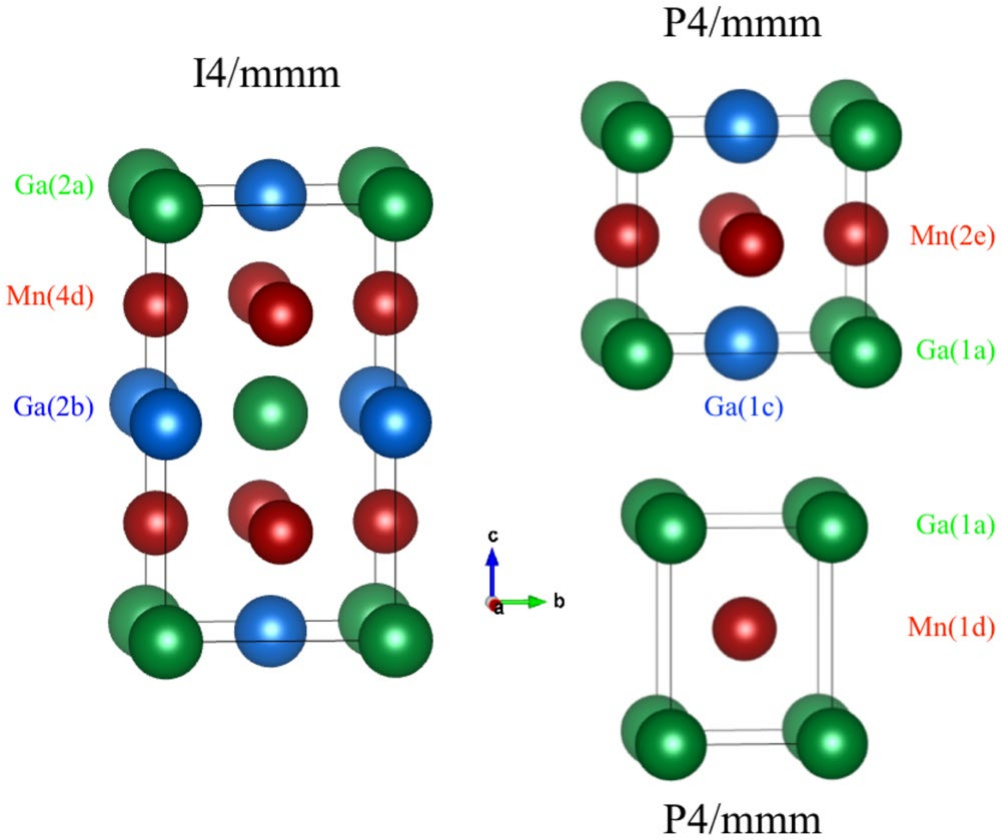
The three tetragonal structures considered here for the Mn_x_Ga system and drawn for stoichiometric MnGa for simplicity. Anti-clockwise from top right: Large cell P4/*mmm* (#123, often denoted as *L*1_0_) generally assumed for MnGa, has two Ga sites and one Mn site; I4/*mmm* (#139, or *D*0_22_) generally assumed for Mn_3_Ga, constructed by stacking two P4/*mmm* cells along the *c*-axis; The simpler reduced-cell P4/*mmm* structure proposed here with one Ga and one Mn site. It is a simplified form of the larger *L*1_0_–P4/*mmm* cell with *c* unchanged and *a*′ = $\sqrt{2}$. In each case the atom sites are identified next to each cell.

Stoichiometric MnGa is reported to adopt the tetragonal P4/*mmm* structure (#123, often denoted as *L*1_0_, shown at the top right of Fig. 1) with *a*~3.9 Å and *c*~3.8 Å, while Mn_3_Ga adopts a closely related tetragonal I4/*mmm* structure (#139, or *D*0_22_, shown at the left of Fig. 1), that is formed by stacking two MnGa cells along the *c* −axis and redecorating. However, in going from P4/*mmm* to I4/*mmm* we pick up an inconvenient selection rule (*l* = 2*n*) and the occupation of the 4*d* site by Mn in the I4/*mmm* structure imposes a further constraint on *l*: *l* = 2*n*, ∀ *h*, *k*. The result of these constraints is that it is essentially impossible to distinguish the P4/*mmm* and I4/*mmm* Mn-Ga structures on the basis of x-ray diffraction data. Indeed, unless there is a severe imbalance in the Mn/Ga occupations of the two Ga sites shown in Fig. 1, the two structures are formally identical: Any packing of a Mn_x_Ga P4/*mmm* cell can be mapped directly onto an I4/*mmm* cell yielding the same (doubled cell) crystal structure and precisely the same diffraction pattern. Even if we assume perfect selectivity, the impact on the diffraction pattern is vanishingly small: Taking a composition of Mn_1.66_ Ga and placing *all* of the excess Mn on the Ga(2a) site in the I4/*mmm* cell (half filling it) changes the intensity of the (002) peak from ~1% of the (112) peak (the strongest in the x-ray diffraction pattern) to ~1.7%. We adopted the I4/*mmm* cell as the starting point for our structural analysis but, as we show below, found that a much simpler structure based on the P4/*mmm* space group with a reduced size unit cell (shown at the bottom right of Fig. 1) provided a consistent fit to our data.

Our interest here is threefold: (1) What is the correct space group and structure for this system? (2) What is the internal packing of the cell? *i.e*. Where does the excess manganese go as we increase *x* from 1? (3) What is the origin of the reduction in magnetisation seen on increasing *x* from 1 in Mn_x_Ga?

The poor x-ray contrast between Mn and Ga means that x-ray diffraction cannot be used to study the distribution of Mn within the cell. Indeed, the x-ray diffraction patterns of Mn_x_Ga are almost indistinguishable from that of a uniform face-centred tetragonal (fct) cell, with the strongest fct-forbidden reflection ((001) for the P4/*mmm* cell) being of order 1% of the intensity of the primary (111) reflection^4^. In addition, magnetic order cannot be studied using conventional x-ray diffraction methods. Therefore, we turn to neutron diffraction where there is almost optimal contrast between Mn and Ga (coherent scattering lengths, *b*_*c*_, are: *b*_*c*_(Mn) = −3.75 fm and *b*_*c*_(Ga) = +7.288 fm), and we are also sensitive to the magnetic ordering. This latter sensitivity is important as the magnetisation of Mn_x_Ga *decreases* with increasing *x* ^3^, suggesting that the additional Mn couples antiferromagnetically (AF) to the ferromagnetic (FM) Mn found in the parent MnGa cell.

Our initial neutron diffraction work on this system confirmed that the moments on Mn atoms that occupied the Ga sites in Mn_x_Ga (1.15 ≤ x ≤ 2.0) were coupled AF to those on the primary Mn site and that this was the origin of the reduction in magnetisation^5^. However, strong correlations between the cell packing and magnetic contributions to the diffraction patterns made an unambiguous determination of the basic structure impossible. Here we eliminate the correlation problem entirely by analysing neutron diffraction patterns taken *above* the ordering temperature of each material where the magnetic contribution is absent, and then using the cell packing information derived from these fits to constrain the analysis of the neutron diffraction patterns taken at ambient temperature. This approach is complemented by using density functional theory (DFT) calculations to investigate possible site preferences for the excess Mn in Mn_x_Ga, and to determine the magnitudes and orientations of the Mn moments on the three possible sites in the (initially assumed) I4/*mmm* cell.

## Experimental Methods

A series of tetragonal Mn_x_Ga (x = 1.15, 1.20, 1.40, 1.50, 1.60 and 1.80) alloys were prepared by induction melting high purity gallium (99.9%) and manganese (99.5%) in an argon atmosphere. To compensate for evaporation losses during melting, an extra 3 wt.% Mn was added to the alloys. The as-cast ingots were annealed in a tubular vacuum furnace at temperatures, T_a_, ranging from 700 K to 900 K for one to seven days and then quenched into ice water. The annealing step is crucial for obtaining single-phase alloys, and the optimal annealing temperature range was found to be quite narrow and depended critically on the composition of the alloy^3^.

**Figure 2:**
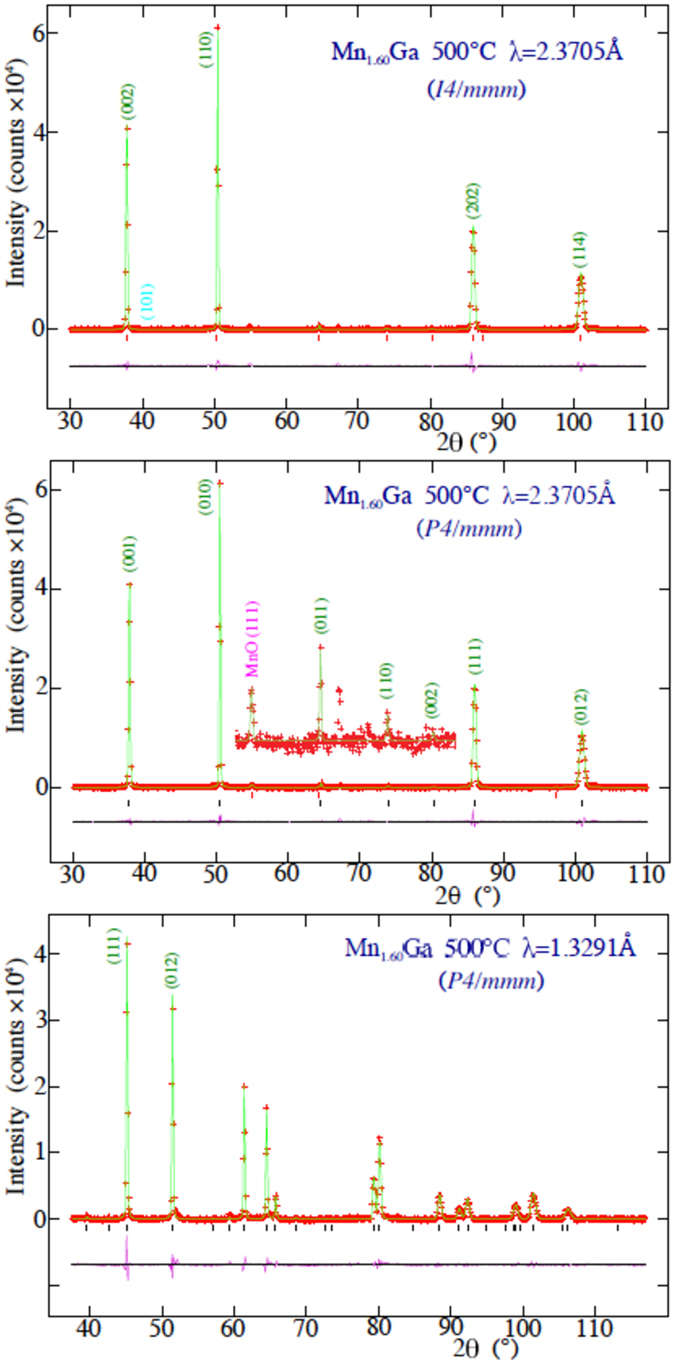
Neutron diffraction patterns for Mn_1.60_Ga taken at 500 °C (*i.e*. above T_C_). The top panel shows the pattern measured using 2.37047(8) Å neutrons and fitted assuming the I4/*mmm* cell. The position of the (101) peak that would be present if the I4/*mmm* cell were the correct choice and there was an imbalance in the Mn occupations of the two Ga sites is also shown (see text for more details). The middle and bottom panels show the data taken with 2.37047(8) Å and (bottom) 1.32905(5) Å neutrons respectively and fitted assuming the small P4/*mmm* cell. The region between 2*θ* = 52° and 2*θ* = 82° has been rescaled by a factor of 20 in the centre panel so that the (111) peak from the 0.25(5) wt.% MnO impurity can bee seen. There is also a small peak from an unidentified impurity at 2*θ* = 67.2°. The solid line in each case is a fit. Bragg markers and a plot of the residuals are also shown. Several Mn_1.60_Ga peaks in the 2.37047(8) Å patterns are indexed, as are the first two in the 1.32905(5) Å pattern (to establish the overlap between the two data sets).

Basic structure and phase purity were confirmed using Cu−*K*_*α*_ x-ray diffraction. Magnetic properties were measured using a Quantum Design Physical Properties Measurement System (PPMS) magnetometer with a maximum magnetic field of 14 T. Neutron powder diffraction experiments were carried out at both ambient and elevated temperatures on the C2 800-wire powder diffractometer (DUALSPEC) at the NRU reactor, Chalk River Laboratories, Ontario, Canada, using neutron wavelengths (*λ*) of 1.32905(5) Å and 2.37047(8) Å to cover the widest range of scattering vectors (0.3 Å^−1^ ≤ *q* ≤ 8.0 Å^−1^). The multi-wire detector covers an 80-degree arc of 2*θ* at one time and the angle ranges used for the two wavelengths were chosen so as to have two strong diffraction peaks in common between the two patterns to facilitate co-refinement (see Fig. 2). For every sample, the patterns at each wavelength were counted so as to have at least 40,000 counts in the strongest diffraction peak.

For the high temperature (non-magnetic) data, shown in Fig. 2, the long and short wavelength diffraction patterns were co-refined to a single structural model for each composition using GSAS^6^/EXPGUI^7^. For each composition, several structural models were tried starting from the I4/*mmm* space group and the final selection was constrained by demanding agreement with the nominal alloy composition. As described below, an evaluation of these fits led us to reject the I4/*mmm* space group as the basis for describing the structure of Mn_**x**_Ga and adopt a simpler reduced-cell P4/*mmm* -based crystal structure for both the structural and magnetic fits.

With the cell symmetry and packing established from the analysis of the high temperature patterns, the ambient temperature patterns (taken at both wavelengths and recorded to the same quality as the high temperature patterns) were then co-refined to determine the Mn moments, subject to the structural constraints derived from the high temperature fits.

To understand the site distribution of the excess Mn at the 2*a* and 2*b* sites in the I4/*mmm* cell, the total energy for Mn_x_Ga (1.0 ≤ x ≤ 2.0) with different occupancy configurations of Mn were calculated by a DFT (density functional theory) method. We set the super-cell with a nominal composition of Mn_4_(Mn_2_Ga_2_) with the four Mn atoms at the 4*d* site and the two Ga atoms at either the 2*a* or the 2*b* site. The remaining two Mn atoms were then placed on the vacant 2*b* or 2*a* site. The QUANTUM ESPRESSO package^8^, in the projected augmented wave (PAW) framework^9^, was employed to perform DFT calculations using the generalized gradient approximation (GGA) of Perdew-Burke-Ernzerhof^10^, ^11^ for the exchange correlation functional. The atomic PAW potentials were adopted from the PS library.1.0 generated by A. Dal Corso^12^. The wave functions were expanded in plane-wave basis sets truncated at a cutoff energy of 60 Ry. Brillouin zone integrations were performed on a 8 × 8 × 8 k-point grid, and Marzari-Vanderbilt broadening^13^ was applied with a smearing width of 5 mRy. The structural degrees of freedom were fully relaxed to obtain optimized structural parameters. In addition, the magnetic moments in Mn_x_Ga have been calculated using a linear muffin-tin orbital method with coherent potential approximation (LMTO-CPA)^14^, ^15^, ^16^.

## High Temperature Results–Cell Symmetry and Packing

Our starting point for the fits to the high temperature neutron diffraction patterns was the body-centred tetragonal I4/*mmm* cell with Mn atoms on the 4*d* site and Ga on both the 2*a* and 2*b* sites giving an initial stoichiometry of MnGa. The two patterns for each composition were fitted by permitting vacancies on the 2*a* and 2*b* sites, partial filling of each site with Mn, or no vacancies with a mix of Mn and Ga on the 2*a* and 2*b* sites. In each case, statistically equivalent quality fits could be obtained, but only the model with the 2*a* and 2*b* sites filled (no vacancies) returned the expected stoichiometry. The other models were therefore rejected.

**Figure 3:**
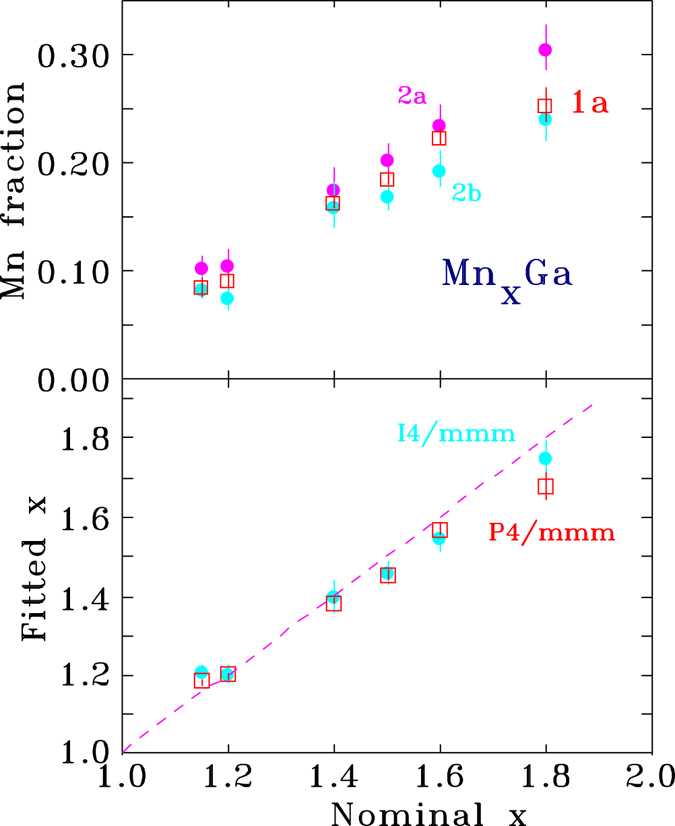
(top) Fitted Mn occupations of the 2*a* and 2*b* sites (assuming an I4/*mmm* cell) compared with the Mn occupation of the 1*a* site assuming the simpler P4/*mmm* cell). (bottom) Stoichiometry derived from the two models. The dashed line has a slope of one and indicates the expected correspondance between the nominal and fitted compositions.

As the top panel of Fig. 3 shows, we appeared to observe a slight systematic preference for Mn to occupy the 2*a* site over the 2*b* site. However, if we reversed the starting point for the fits by exchanging the occupations of the 2*a* and 2*b* sites, we could obtain a fit with the same χ^2^ and precisely the same occupations (to four digits) but with Mn favouring the 2*b* site over the 2*a* site. Examination of simulated diffraction patterns with a stronger 2*a*:2*b* bias revealed that an occupation imbalance led to appearance of the (101) peak (near 2*θ* = 40° in the long wavelength pattern – see upper panel of Fig. 2) and some minor changes in the intensity of the (002) and (110) peaks to the left and right of the (101) peak respectively. The (101) peak was absent from all of our measured patterns and it therefore appears that the 2*a*:2*b* imbalance was an artefact of the fitting program chasing noise.

**Figure 4:**
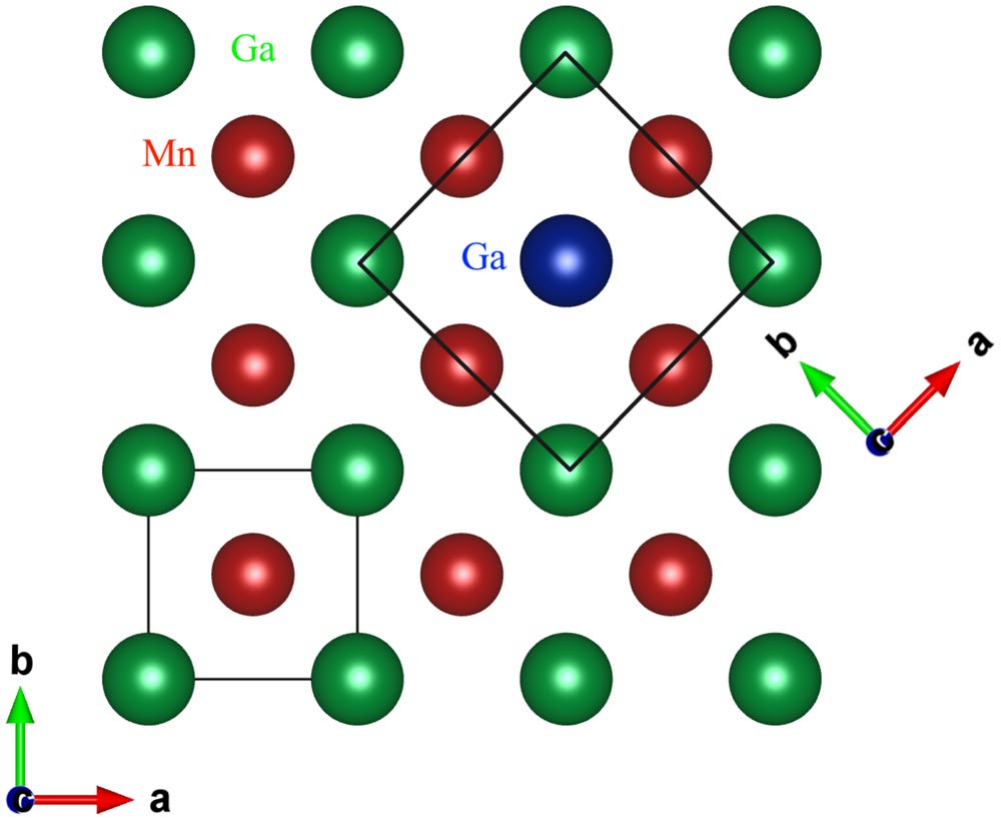
The basal planes for the small (bottom left) and large (top right) P4/*mmm* cells showing the relationship between the *a*-axes in the two cases. The smaller-cell P4/*mmm* basal plane is rotated by 45° and reduced in size by a factor of $\sqrt{2}$ with respect to the large-cell P4/*mmm* basal plane. See Fig. 1 for views perpendicular to the *c*-axis.

Constraining the occupations of the 2*a* and 2*b* sites to be the same had no impact on the quality of the final fit and still returned the expected stoichiometry. This observation led us to consider the possibility that the 2*a* and 2*b* sites were not actually distinct: *i.e*. that the space group was incorrect. A search for possible alternative space groups led us back to the simple tetragonal P4/*mmm* space group (#123) but with the reduced-size unit cell originally proposed by Bither and Cloud^17^. Figure 4 shows the relationship between the basal planes of the large and small cell P4/*mmm* structures. Placing the Mn atoms in the 1***d*** site (located at $\frac{1}{2}$ $\frac{1}{2}$ $\frac{1}{2}$) and the Ga atoms in the 1*a* site (located at 0 0 0) yields precisely the same packing as our I4/*mmm* starting point, and the *same* calculated diffraction pattern. The (101) peak (in the I4/*mmm* cell) that could have signalled a difference in Mn occupation between the “two” Ga sites is forbidden, consistent with the sites now being equivalent. Refinements from this starting point yield fits that were fully equivalent to those obtained using the I4/*mmm* cell and returned the expected stoichiometry, but required fewer fitting parameters. Fitted patterns for the Mn_1.60_Ga sample taken at 500 °C and using the smaller P4/*mmm* cell are shown in Fig. 2.

To further investigate whether the 2*a* and 2*b* sites in the I4/*mmm* cell were indeed equivalent, we carried out DFT calculations looking at the energy associated with placing Mn on either the 2*a* or the 2*b* site. Our calculations showed that the binding energy for the excess Mn at the 2*a* site is precisely the same as that for Mn at the 2*b* site (−4.08 eV/atom). We therefore conclude that the two sites are indeed fully equivalent and that the P4/*mmm* space group is the correct one to use for Mn_x_Ga. As a final check, we confirmed that the energies associated with substituting Mn at each of the two Ga sites in the large-cell P4/*mmm* structure were also the same. The revised structural description is simpler (only two crystallographic sites are occupied) and the stoichiometry for the end-member (MnGa) is obtained directly.

The Mn occupation of the 1*a* site and the resulting stoichiometry are plotted in Fig. 3. These results form the starting point for our analysis of the magnetic ordering in the ambient temperature patterns.

## Ambient Temperature Results – Magnetic Ordering

**Figure 5:**
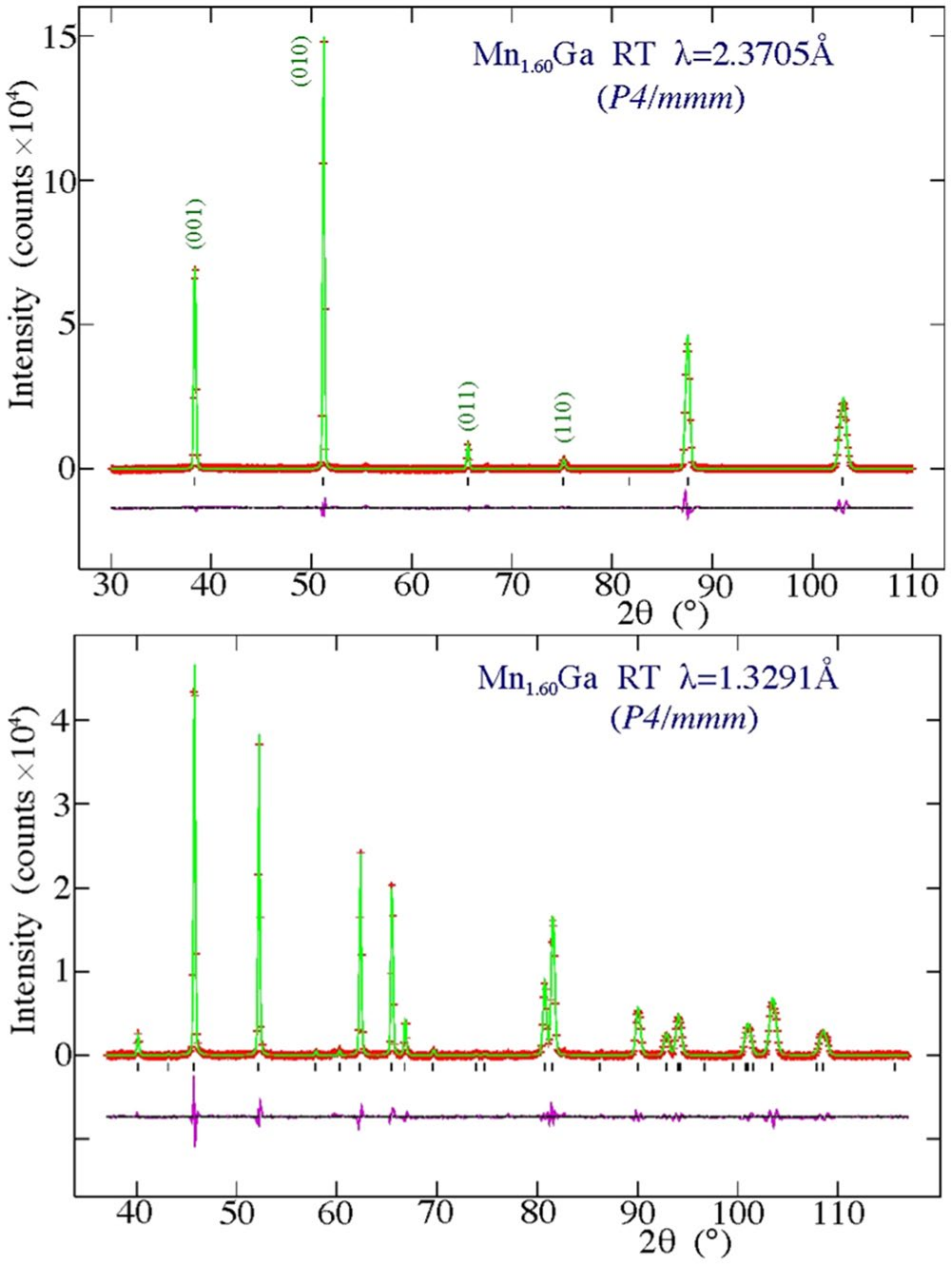
Neutron diffraction patterns for Mn_1.60_Ga taken at ambient temperature (*i.e*. below T_C_) using (top) 2.37047(8) Å and (bottom) 1.32905(5) Å. The solid line in each case is a fit to the P4/*mmm* magnetic and structural model described in the text. Bragg markers and a plot of the residuals are also shown. The two strongest magnetic contributions at (011) and (110) are identified on the 2.37047(8) Å pattern.

The most striking feature of the ambient temperature patterns for Mn_1.60_Ga shown in Fig. 5 is how little they change in comparison with the 500 °C patterns shown in Fig. 2. The most visible changes are the increase in the intensity of the (010) reflection relative to the (001) and some very small increases in intensity at the (011) and (110) positions. This immediately serves to underline the critical need both for high quality patterns and to establish the locations of the excess Mn atoms within the cell before proceeding to a determination of the magnetic structure. The magnetic and crystallographic cells are the same size (no cell doubling occurs when the magnetic order develops) so the magnetic scattering appears only at nuclear-allowed positions. There is significant cross-talk between the scattering contributions from the cell packing and the magnetic ordering, and there are many different ways to play off these contributions^5^. However, by determining the Mn locations above T_C_ we sidestep this problem entirely, and can proceed to determining the magnetic moments free of ambiguity.

The magnetic structure used as a starting point was based on the P4/*mm*′*m*′ magnetic space group that allows for *c*-axis FM ordering of the Mn moments on the 1*d* site. This magnetic group also permits *c*-axis ordering at the 1*d* site where we have shown that the excess Mn substitutes for Ga. Only collinear magnetic structures were considered here. The magnetic space group adopted for the analysis here is essentially equivalent to the I4/*mm* ^*’*^ *m* ^*’*^ magnetic space group used previously when the I4/*mm* ^*’*^ *m* ^*’*^ structure was being considered^5^.

**Figure 6:**
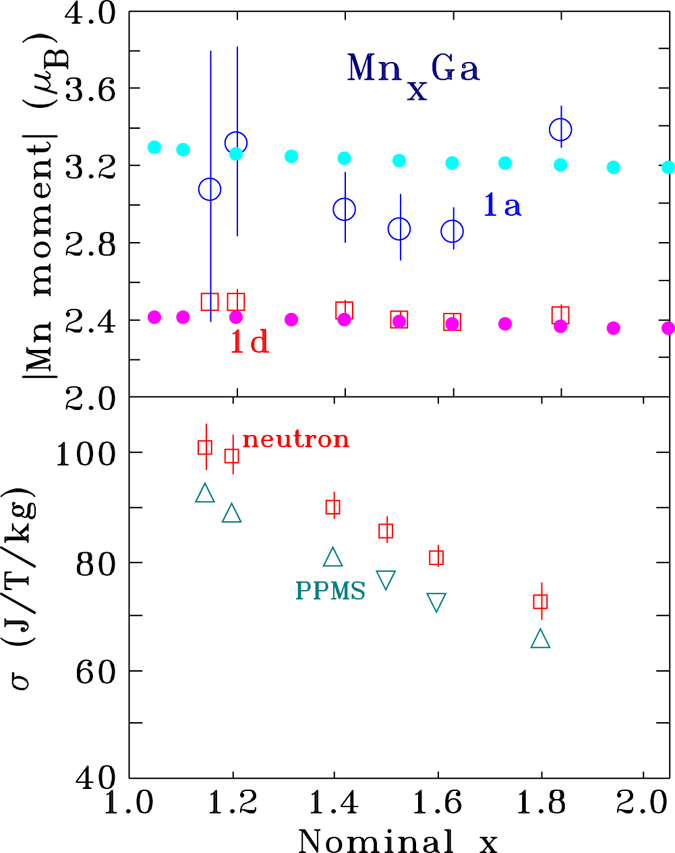
(top) The open symbols show the fitted Mn moments for the two sites in the P4/*mmm* Mn_x_Ga structure. The moment of the manganese on the 1*d* site is essentially constant at 2.45(3) *μ*_B_/Mn while a slightly larger antiparallel moment of ~3 *μ*_B_/Mn appears on Mn atoms that occupy the 1*a* site. Also shown as solid points are the corresponding moments calculated using DFT. (bottom) Net magnetisation derived from the neutron scattering fits (neutron) compared with values obtained from bulk magnetisation data (PPMS).

**Figure 7:**
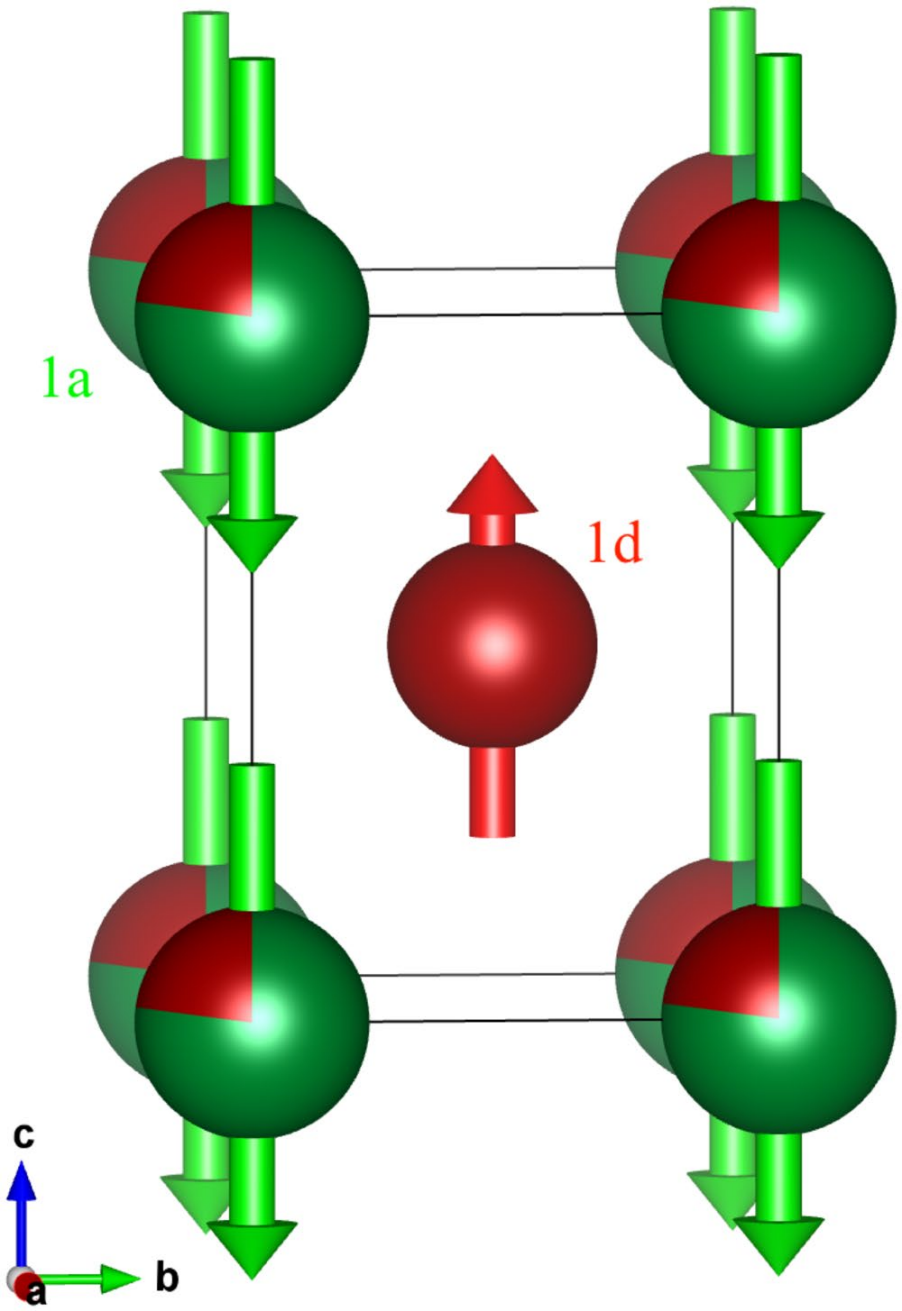
Magnetic structure for Mn_x_Ga deduced from our fits to the neutron diffraction data. The Mn moments on the manganese that partially replaces gallium on the 1*a* site are slightly larger and antiparallel to those on Mn that occupies the 1*d* site.

For all compositions we find that the Mn atoms that replace Ga atoms on the 1*a* site have moments that couple antiparallel to those on the primary 1*d* site, broadly consistent with our earlier analysis^5^. In addition, we find that the size of the Mn(1*d*) moment is essentially independent of composition at 2.45(3) *μ*_B_/Mn. The situation at the 1*a* site is somewhat less clear as the Mn occupation of this site never exceeds 0.25 and for the two lowest excess Mn samples (*x* = 1.15 and 1.20) the Mn occupation of the 1*a* site is only ~0.1. This leads to rather large uncertainties on the fitted moment, however it appears to be broadly constant, and at 3.0(1) *μ*_B_/Mn, it is clearly larger than that on the 1*d* site. These results are plotted on the upper panel of Fig. 6. Comparison of the derived magnetisation with that measured by bulk magnetometry (lower panel of Fig. 6) shows that the neutron results track well with the bulk data but appear to be about 10% higher. The origin of this offset is not understood, and it may reflect some incomplete saturation in the bulk data. We can however rule out canting of the moments. Strictly speaking, occupation of the 1*d* and 1*a* sites in the P4/*mmm* space group is only compatible with purely *c*-axis ordering, but leaving this issue of principle aside, any planar (*ab*−) component to the magnetic order would almost certainly lead to a severe mis-fit to the intensity of the (002) peak, especially given the significant magnetic contribution to the (110) peak. Any long-period modulation of the order (spirals, cones *etc*.) would be expected to contribute both magnetic satellites and a low-angle peak from the new periodicity. Neither are seen (see Fig. 2 of Rejali *et al*.^5^ for data down to 0.3 Å^−1^). It is clear from our fits to the magnetic structure that the decline in bulk magnetisation with increasing Mn in Mn_x_Ga is the result of antiparallel Mn moments on the 1*a* (Ga) sites (shown in Fig. 7).

Calculated moments for the two sites derived from our DTF work are shown for comparison as solid symbols on the upper panel of Fig. 6. The agreement is remarkably close. For the Mn on the 1*d* site we observe a slight (~2.5%) decline over the studied range, with an average value of 2.40(2) *μ*_B_/Mn, while for the Mn on the 1*a* site we observe a similar, ~3%, decline with increasing Mn content and an average value of −3*:*24(3) *μ*_B_/Mn. The former moment is in perfect agreement with the experimental value, while the latter value is only slightly outside experimental error for the average value and well within the experimental scatter (Fig. 6).

## Conclusions

Neutron powder diffraction patterns measured above and below T_C_ have been used to determine the site occupancies and magnetic properties of a series of Mn_x_Ga (1.15 ≤ x ≤ 1.8) alloys. Both our high temperature diffraction patterns and DFT calculations show that there is no difference between the the 2*a* and 2*b* sites in the commonly used I4/*mmm*–based (*D*0_22_) structure, or equivalently, 1*a* and 1*c* sites in the large-cell P4/*mmm* (*L*1_0_) structure. We therefore propose a much simpler crystal structure based on the I4/*mmm* space group with a reduced unit cell, originally suggested by Bither and Cloud^17^. We find that the excess Mn in Mn_x_Ga replaces some of the Ga on the 1*a* site, and with the crystal structure as a constraint we are able to make an unambiguous determination of the magnetic structure and Mn moments. Antiparallel ordering between the 2.45(3) *μ*_B_ Mn moments on the 1*d* site and the 3.0(1) *μ*_B_ moments associated with the excess Mn on the 1*a* site leads to the observed decline in bulk magnetisation. These results are in full, quantitative, agreement with both DFT calculations and bulk magnetisation measurements.

If hard magnets are to be developed based on the Mn_x_Ga system, it will be necessary to find something that will substitute for Ga on the 1*a* site but that either carries a much smaller moment than the Mn (without causing a loss of anisotropy) or couples parallel to the Mn moments on the 1*d* site. Given the remarkable agreement between our experimental data and the DFT calculations, it would appear that a preliminary search using DFT calculations could reliably identify promising candidates and so greatly reduce the synthetic effort associated with the development of new materials in this system.
